# Supplementary figures and images for: On the origin of mitochondria: a multilayer network approach
Source: PeerJ. 2023 Jan 6;11:e14571. doi: 10.7717/peerj.14571 (PMC9828282; doi:10.7717/peerj.14571)

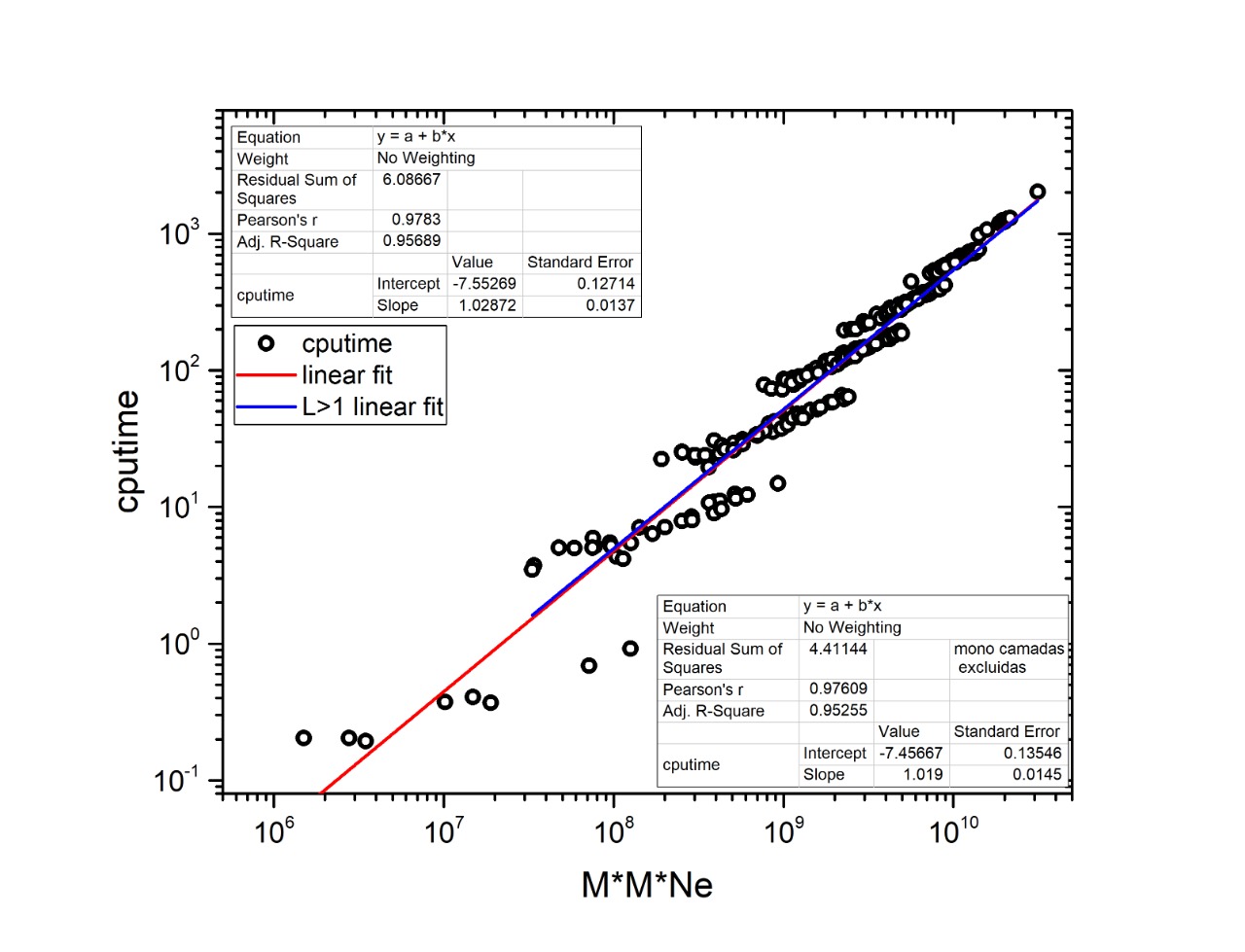

Supplement: Supplemental Information 1 — The analysis of the algorithmic complexity of MultiNG approach (Github: https://github.com/randradeufba/MultiNG), including the regression analysis, the statistical test, its theoretical linear fit, and CPU time. [file peerj-11-14571-s001.jpeg]

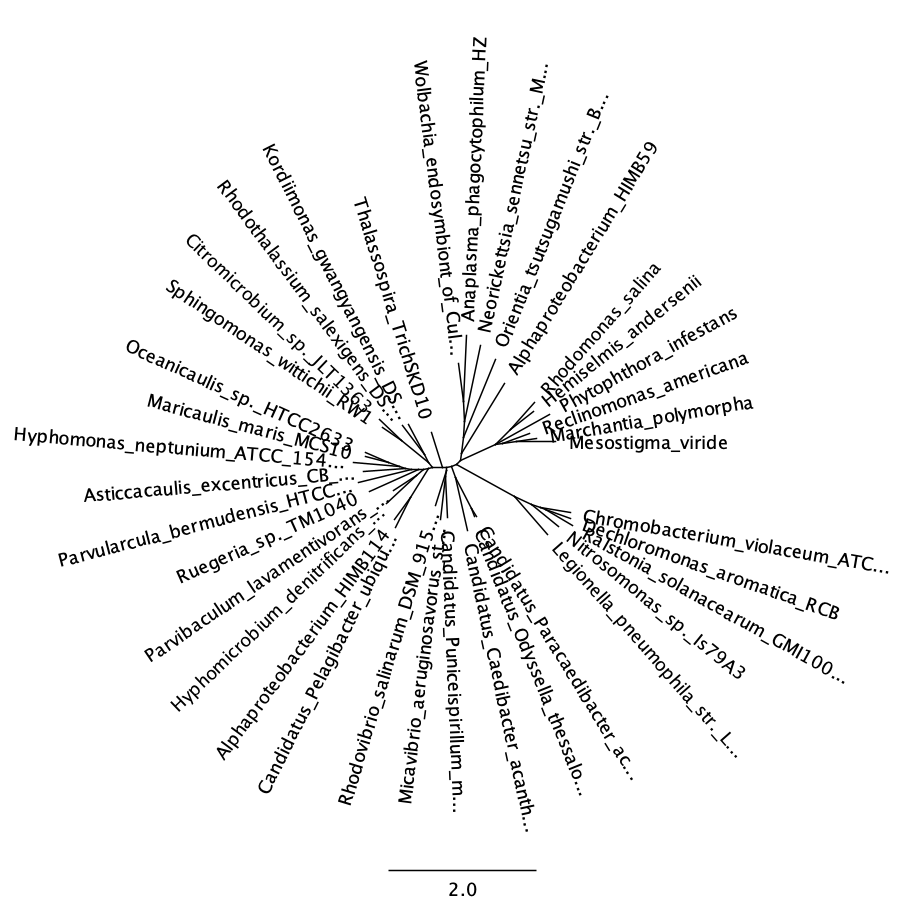

Supplement: Supplemental Information 2 — The consensus tree of Bayesian tree-based method, using the best-fit model of protein according to AIC (LG+I+G+F), shows results similar to those proposed by Wang & Wu (2015) (supporting Rickettsiales-sister hypothesis). [file peerj-11-14571-s002.png]
