# Supplementary material for: On the origin of mitochondria: a multilayer network approach
Source: PeerJ. 2023 Jan 6;11:e14571. doi: 10.7717/peerj.14571 (PMC9828282; doi:10.7717/peerj.14571)
Supplement: Supplemental Information 7 [file peerj-11-14571-s007.docx]

Supplementary Material

By analyzing the bifurcation events that took place within community C3, we identified the early branching, as revealed by the sequential removal of edges in the multiplex, of 10 out of the 17 Rickettsiales sequences included in the dataset (Figure 3 in main text). This result is also confirmed when higher values for sigma are used to disclose communities (Cob: 70%; Cox2: 61%; Cox3: 67%; Nad1: 72%; Nad4: 68%; Nad5: 67%; Nad6: 67%; Nad9: 66%). In Supplementary Figure 1 we further try to improve the resolution of the relationships within the subclusters, confirming the grouping of early-branching Rickettsiales, of most Rhodobacterales and that of Rhizobiales sequences. Taken together, these results provide strong support toward the divergence of Rickettsiales before the diversification of the remaining alphaproteobacterial subgroups.

Explanation of Supplementary Figure M1

Supplementary result to Figure 3 in the main text: dendrogram obtained by applying the MultiNG algorithm to the weighted layer multiplex network at larger values of σ*_th_* (Cob: 70%; Cox2: 61%; Cox3: 67%; Nad1: 72%; Nad4: 68%; Nad5: 67%; Nad6: 67%; Nad9: 66%). Although the initial number of isolated branches is larger than that of Figure 3, for the sake of a better identification the sequences are distributed into communities C0-C3 according to identification defined in that figure. For the same reason, the 10 early diverging Rickettsiales within C3 are drawn in red. Contrary to what was observed in Figure 3, the dendrogram further discloses eight well defined sub-clusters within the C3, and the highest modularity value Q = 0.9461 just before a series of important divergences leading to the subgroups. Some of them are characterized by the presence of most Rhodobacterales and Rhizobiales sequences.

Description table 1 supplementary

Species included in the work, their taxonomic information and community members, shown in figure 2 (main dendrogram) revealed during the evaluation of the network structure.

Description table 2 supplementary

Species included in the work, their taxonomic information and community members and subcommunities (if any), shown in complementary figure 1 (supplementary dendrogram) revealed during the network structure assessment.

Description table 3 supplementary

Species included in the work, abbreviated nomenclature, representation of the number of each species.
